# Total gastrectomy for locally advanced cancer: the pure laparoscopic approach

**DOI:** 10.1093/gastro/got005

**Published:** 2013-03-28

**Authors:** J.S. Azagra, M. Goergen, L. Arru, O. Facy

**Affiliations:** Department of General and Mini-Invasive Surgery, Centre Hospitalier de Luxembourg

## INTRODUCTION

Total gastrectomy is the treatment of choice for adenocarcinoma of the upper and middle third of the stomach resected with curative intent. The laparoscopic approach allows satisfactory exploration of the peritoneal cavity and optimizes staging in borderline T3 or T4 tumours in patients affected by locally advanced tumours or intraperitoneal carcinomatosis. Laparoscopy can eliminate unnecessary laparotomies in 10% of patients affected by these conditions with formal contraindications for resection [1]. Complete resection of the stomach associated with D2 lymph node dissection is also performed using a currently well-established technique [2, 3]. The specificity of laparoscopic gastric resection for cancer is that the stomach and the great omentum are withdrawn separately.

Reconstruction of the digestive tract is more complex and requires a variety of techniques (supra-umbilical mini-laparotomy, Orvil® technique, enlarging a port-site for passage of a circular stapler, mechanical side-to-side anastomosis, etc), but none of these has become the ‘gold standard’ [4–7]. This explains the difficulties encountered in promoting the widespread use of minimally invasive resection in western countries. Scientific societies insist on the need for prospective studies to establish the place of laparoscopy for gastric cancer (prophylactic gastrectomy for CDH-1 related gastric cancer, <T3 tumours, palliative gastrectomy) [4].

Here we present our technique for total resection of the stomach and D2 lymph node dissection, which allows the manual creation of a feasible, safe, tension-free and effective oesophago-jejunal anastomosis. It can be performed by any surgeon familiar with laparoscopic surgery and the principles of oncological resection. The cost is also relatively low because neither a circular stapler nor other special equipment is required. Finally, the incision for extraction of the specimen can be placed in any area of the abdomen (usually through a suprapubic incision in our practice).

## PATIENT POSITION AND PLACEMENT OF THE TROCARS

**Fig. 1:**
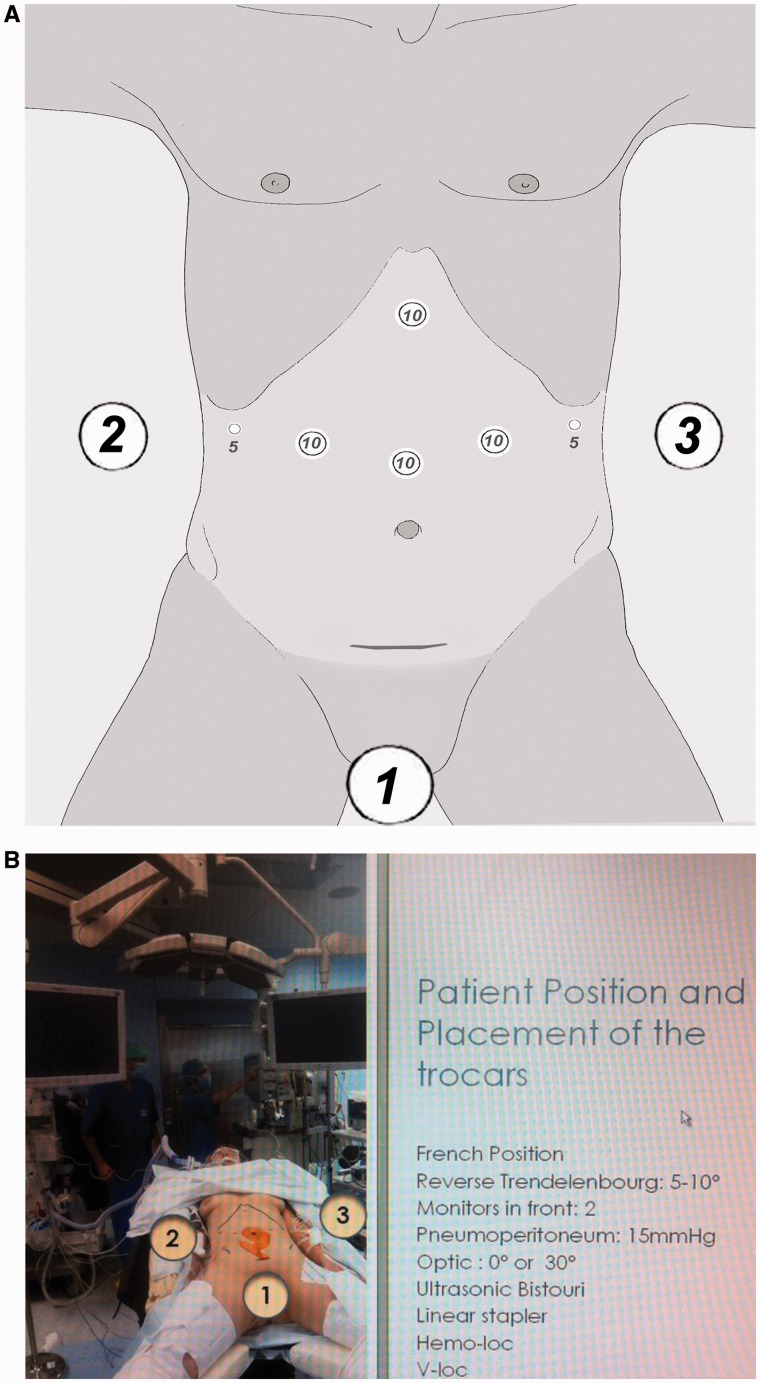
(A) Placement of the trocars and position of the surgeons (1, 2 and 3). (B) Patient position and surgical set-up.

The patient is placed in the ‘split leg position’, with an inclination of 5–10° in the reverse Trendelenburg position (Fig. 1). The first surgeon stands between the legs of the patient, with an assistant on each side. Four 10 mm trocars are placed in the upper part of the abdomen: two in the xypho-umbilical line and two in the mid-clavicular right and left lines. Two 5 mm trocars are placed, one each in the right and left hypochondrium. The 0° telescope is placed in the supra-umbilical trocar for the submesocolic surgery and then in the subxyphoidal trocar during the supramesocolic surgery for total gastrectomy with D2 lymphadenectomy.

## GASTRECTOMY AND D2 LYMPHADENECTOMY

### Infra-pyloric lymphadenectomy (Fig. 2)

After thorough exploration of the peritoneal and hepatic areas, the great omentum is sectioned 2 cm below the gastro-epiploic vessels at the level of the antrum. Access to the omental cavity is widened to the left to establish the loco-regional spread. The gastro-splenic vessels are sectioned to the left gastro-phrenic ligament (group 4). The gastro-colic ligament is cut using the ultracision® device (Ethicon Endosurgery). The right gastro-epiploic vessels are exposed with traction of the antrum to the top (by grasping the posterior wall of the stomach) and ligated from their origin on the gastro-duodenal artery and the gastro-colic vein. The artery is dissected forwards to the duodenum to the point where it arises from the hepatic artery (group 6).

**Fig. 2:**
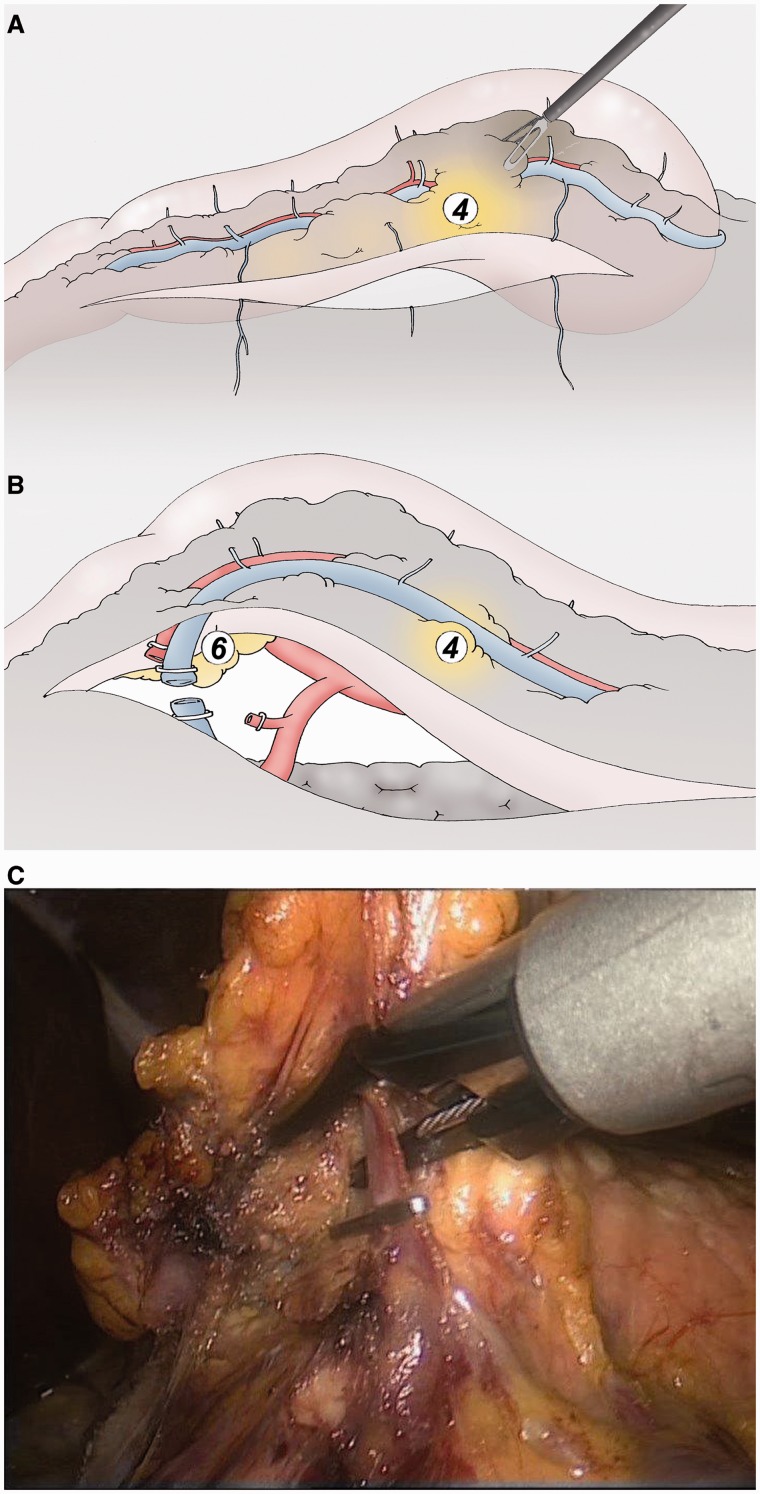
(A) The great omentum is sectioned 2 cm below the gastro-epiploic vessels to access to the omental cavity and to separate the gastric specimen from the omentum (group 4). (B, C) The right gastro-epiploic vein and artery are separately ligated at their origins (group 6).

### Supra-pyloric lymphadenectomy (Fig. 3)

The hepato-duodenal ligament is cut above the duodenum and the retro-duodenal dissection is joined. The right gastric artery is ligated at its origin on the hepatic artery (group 5). The first part of the duodenum is sectioned 2 cm after the pylorus, using a blue stapler. The lesser omentum is cut starting from the lower side of the liver up to the right side of the oesophagus (group 3). Attention must be paid to a possible left hepatic artery arising from the left gastric artery which, if large, must be dissected free and conserved.

**Fig. 3:**
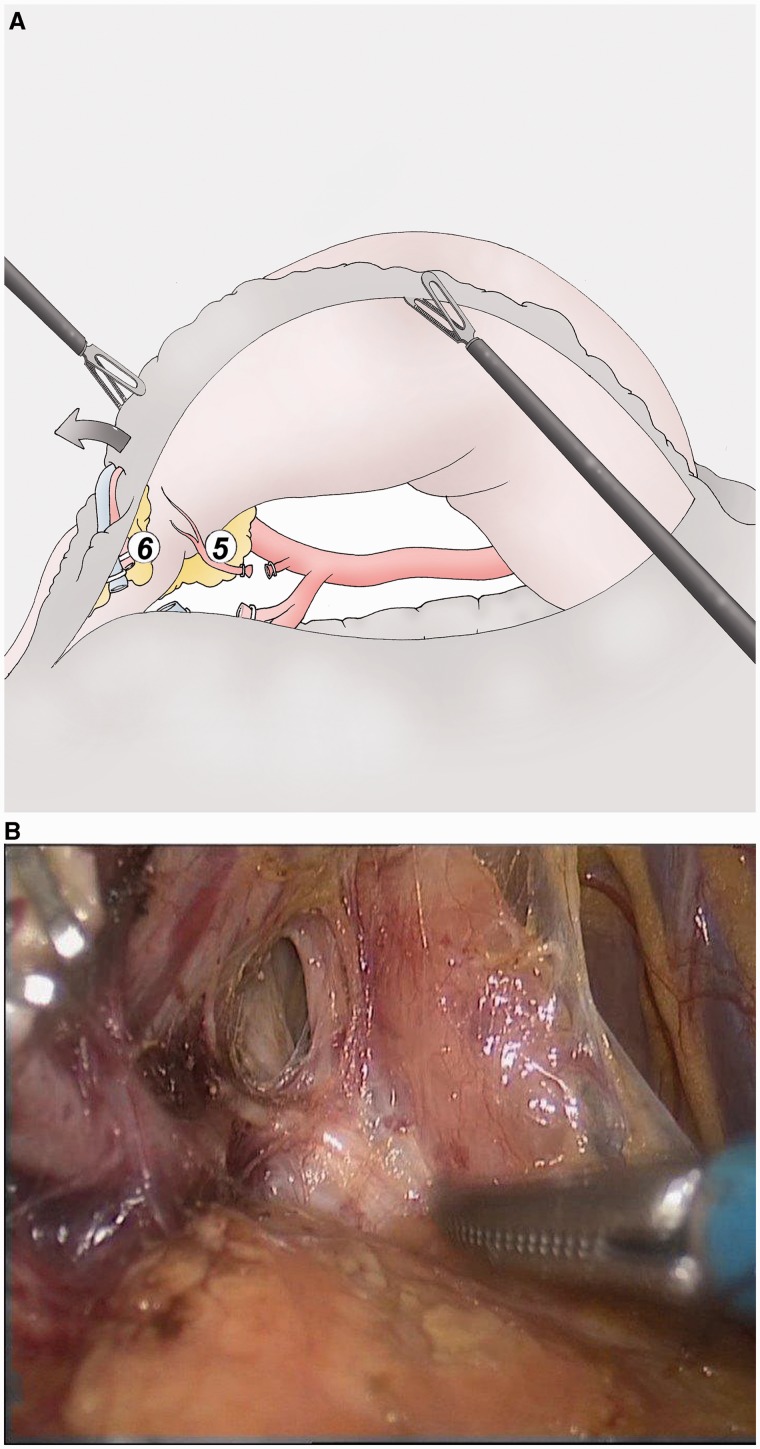
(A, B) The gastroduodenal artery is dissected along the posterior face of duodenum until its origin from the hepatic artery. The right gastric artery is then ligated (group 5).

### Hepatic and splenic lymphadenectomy (Fig. 4)

The gastric specimen, separated from the omental specimen, is placed in the left hypochondrium. The separation of the specimens improves the manipulation of the stomach for an appropriate lymphadenectomy. The hepatic artery is dissected to the celiac trunk at the top of the pancreas (group 8). The splenic artery is dissected for the first 3 cm from the celiac trunk (group 9), as is the origin of the left gastric artery. The left gastric vein is ligated at the top of the pancreas using an unresorbable clip (Hem-o-lok®, Teleflex, USA). The left gastric artery is ligated at its origin on the celiac trunk using the same device (group 7).

**Fig. 4:**
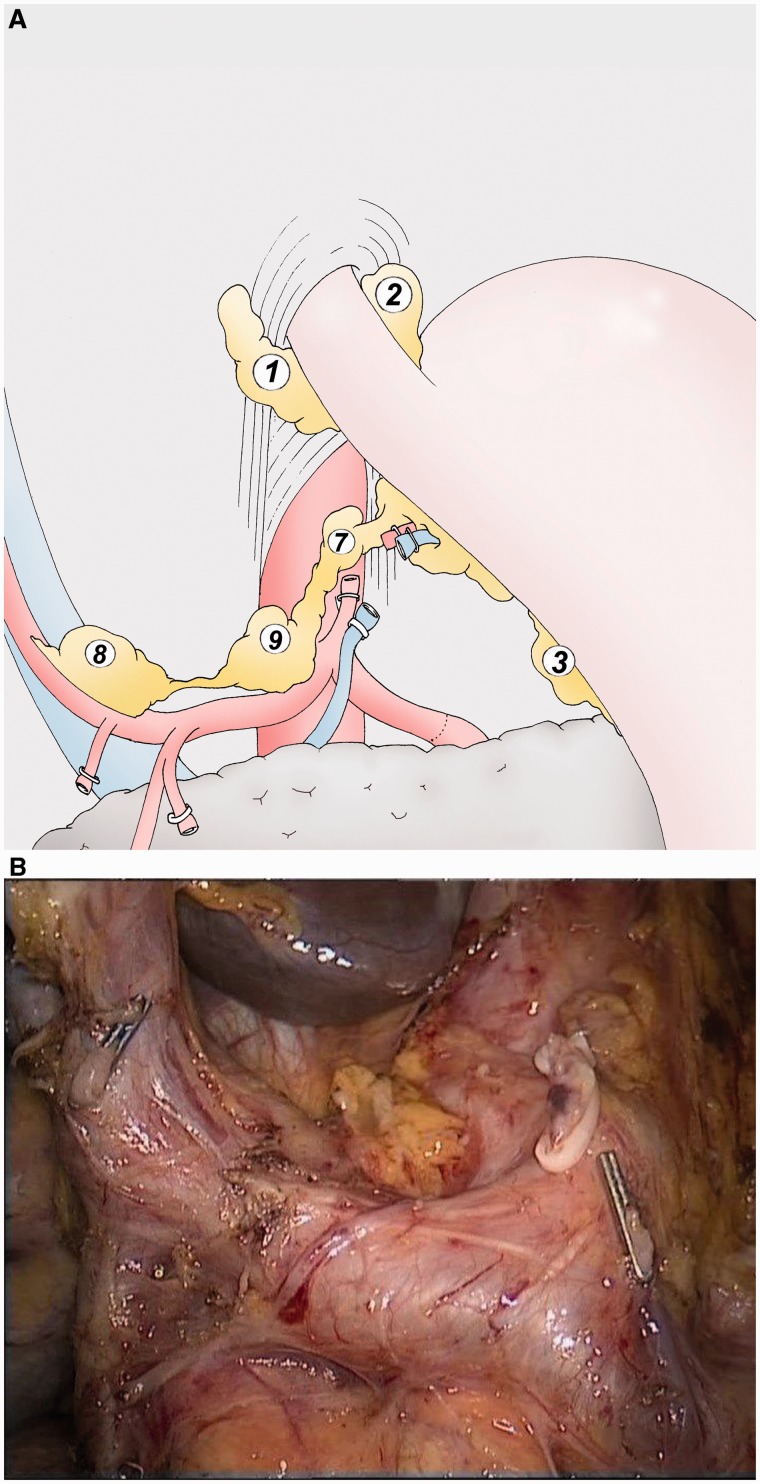
(A) The lymphadenectomy of the group 8 is performed with the dissection of the common hepatic artery as far as the celiac trunk (group 9). The left gastric vein is sectioned to access to the left gastric artery and to cut it (group 7). The splenic artery is dissected for the first 3 cm. (B) Final aspect of the lymphadenectomy of the groups 7, 8 and 9. The right gastric artery is sectioned above standard clip, as well as the left gastric vein. The left gastric artery is sectioned above Hem-o-lock®.

### Para-cardial lymphadenectomy (Fig. 5)

The lymphadenectomy is continued along the aorta to the left and right diaphragm (groups 1 and 2). The lymphadenectomy of the inferior mediastinum can be performed easily when necessary. Its limits are the aorta (posterior), the pericardium (anterior) and the left and right pleura (lateral).

**Fig. 5:**
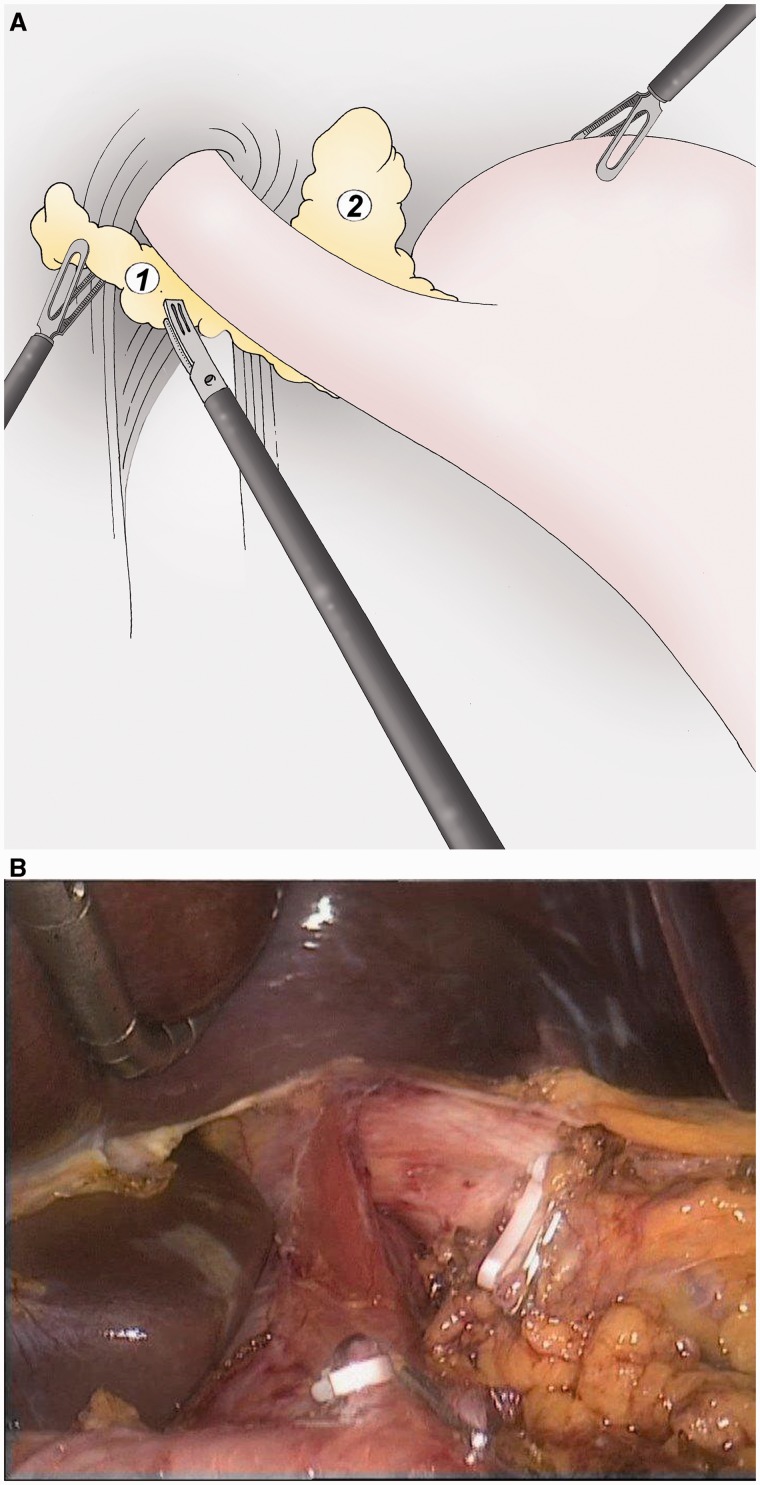
(A) The dissection continues from back to the top along the aorta to the diaphragm and the oesophagus (groups 1 and 2). (B) The dissection rises up along the aorta to the diaphragm and the oesophagus (group 1).

### Abdominalization and section of the distal oesophagus (Fig. 6)

The oesophagus, widely freed from the lower mediastinum (including section of both vagus nerves) is sectioned transversely 2 cm above the cardia with a flexible automatic stapler. The specimen is then placed in the pelvis for suprapubic extraction, protected in a skirt and a frozen section of the proximal section.

**Fig. 6:**
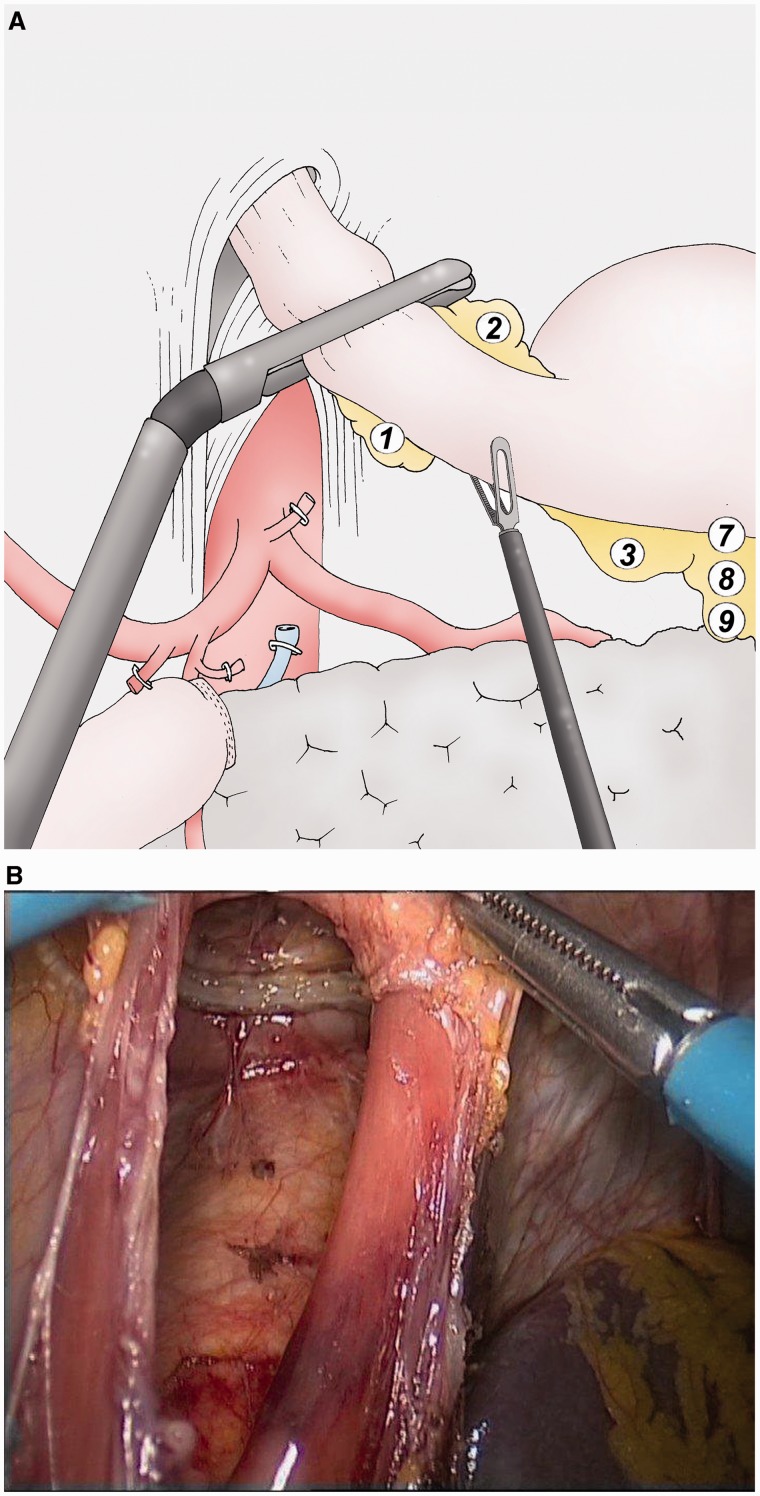
(A) Section of the oesophagus 2 cm above the gastroesophageal junction using an Echelon Flex® 60 mm blue. (B) After the stapler section, the oesophagus lifts up in the mediastinum. A mild pressure using a Faucher tube maintains it in the abdomen.

### Hepatic pedicle lymphadenectomy (Fig. 7)

This part of the D2 lymphadenectomy is more easily performed when the gastric specimen is removed. The left side of the hepatic pedicle is dissected together with the contact of the hepatic artery and portal vein (group 12a).

**Fig. 7:**
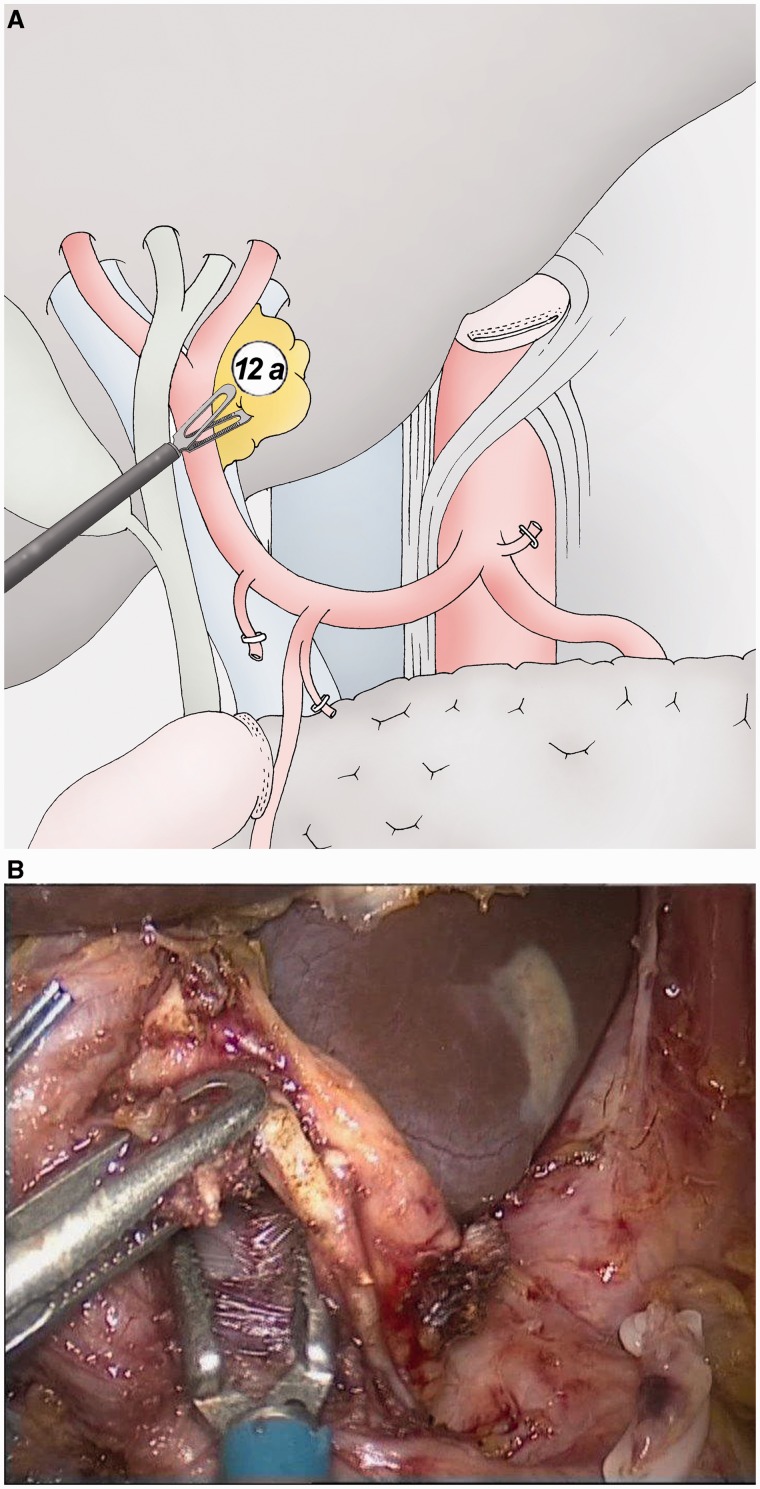
(A) Separated lymphadenectomy of the group 12a (left part of the hepatic pedicle). (B) The left side of the hepatic pedicle is dissected along the hepatic artery and the portal vein (group 12A).

### Omentectomy (Fig. 8)

There is no oncological evidence for the removal of the greater omentum but the omentectomy is widely associated with gastrectomy and D2 lymphadenectomy for cancer [8]. It can be moved more easily following initial separation from the stomach. It is sectioned using the ultracision® device while the omentum is lifted upwards by the assistants. The specimen is placed in a skirt for suprapubic extraction.

**Fig. 8:**
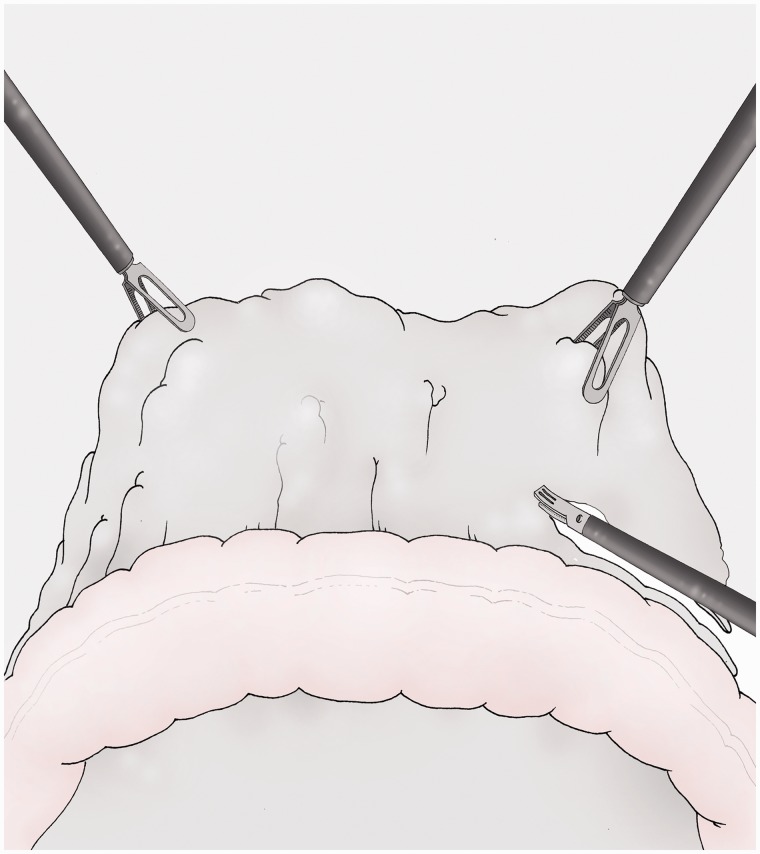
The great omentum is separately removed after the gastric resection using traction from left and right by the assistants.

## RECONSTRUCTION

### Jejunojejunostomy (Fig. 9)

Our preference is to start the anastomosis by the jejunojejunostomy but it could be performed after the oesophago-jejunostomy. Between 20–40 cm from the ligament of Treitz, the jejunal loop that reaches the hiatus with the least traction is cut with the linear stapler. Then the mesentery and first vascular arcade are sectioned using the Ultracision® device. The alimentary limb is measured at 60 cm and a 6 cm mechanical side-to-side jejunojejunal anastomosis is performed using the linear stapler. The inlet is closed by a suture of a 3/0 V-loc® (Covidien, United Kingdom). The internal mesenteric orifice is itself closed with a non-absorbable suture thread.

**Fig. 9:**
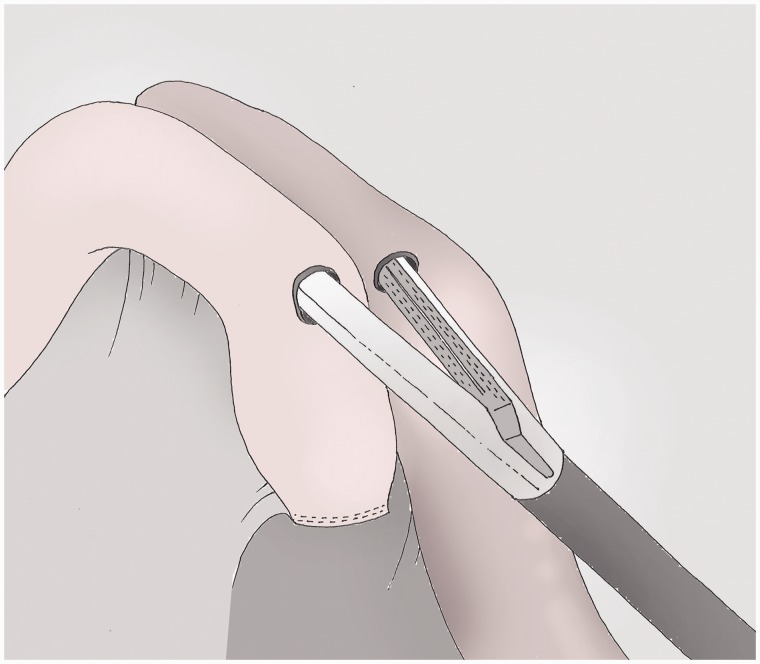
Side-to-side jejunojejunostomy using an Echelon Flex® 60 mm blue.

### Transmesocolic passage of the alimentary loop (Fig. 10)

The transverse mesocolon is opened 1 cm above the ligament of Treitz (in its thinnest part). The alimentary loop is lifted gradually over about 15 cm. The mesocolic defect is then closed with a running suture mounted on the holder.

**Fig. 10:**
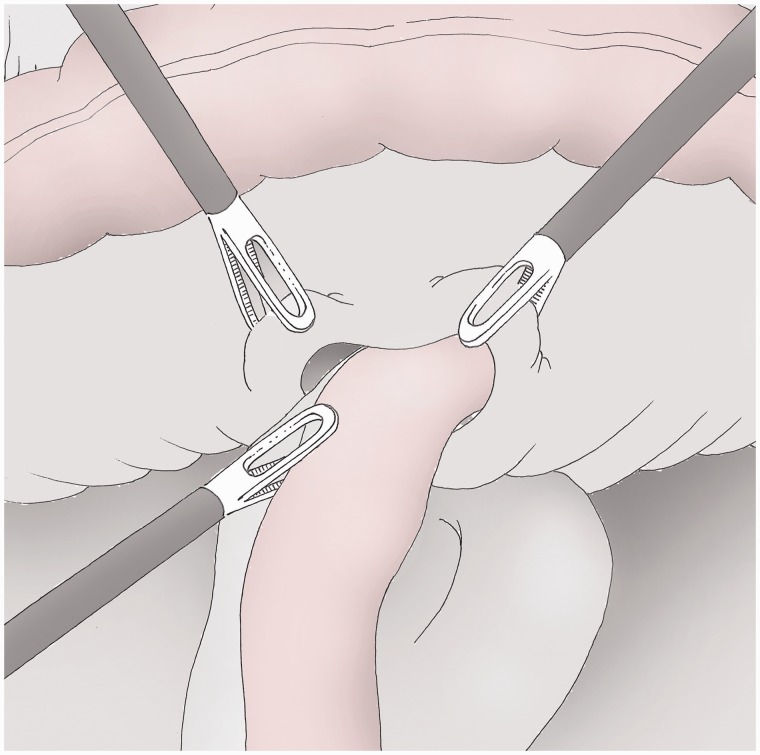
The mesocolon is tracked by the assistants and opened above the ligament of Treitz. The alimentary loop is lifted up through this defect, which is then closed using a non-resorbable suture.

### The oesophago-jejunal anastomosis (Fig. 11) [9]

The oesophagus is held in the abdominal position with the help of mild trans-oral continuous pressure, applied using an atraumatic Faucher tube (with a diameter of at least 33 French). The aid to the right of the operator holds the alimentary loop by pulling gently towards the left hypochondrium. The posterior layer of the anastomosis starts left of the oesophageal staple line and is achieved by a locking suture of a 15 cm V-Loc® (Covidien, United Kingdom). The use of this thread should not be considered as offsetting the inexperience of the surgeon in laparoscopic suturing, but to improve it in an area where exposure and continuous traction are often difficult.

**Fig. 11:**
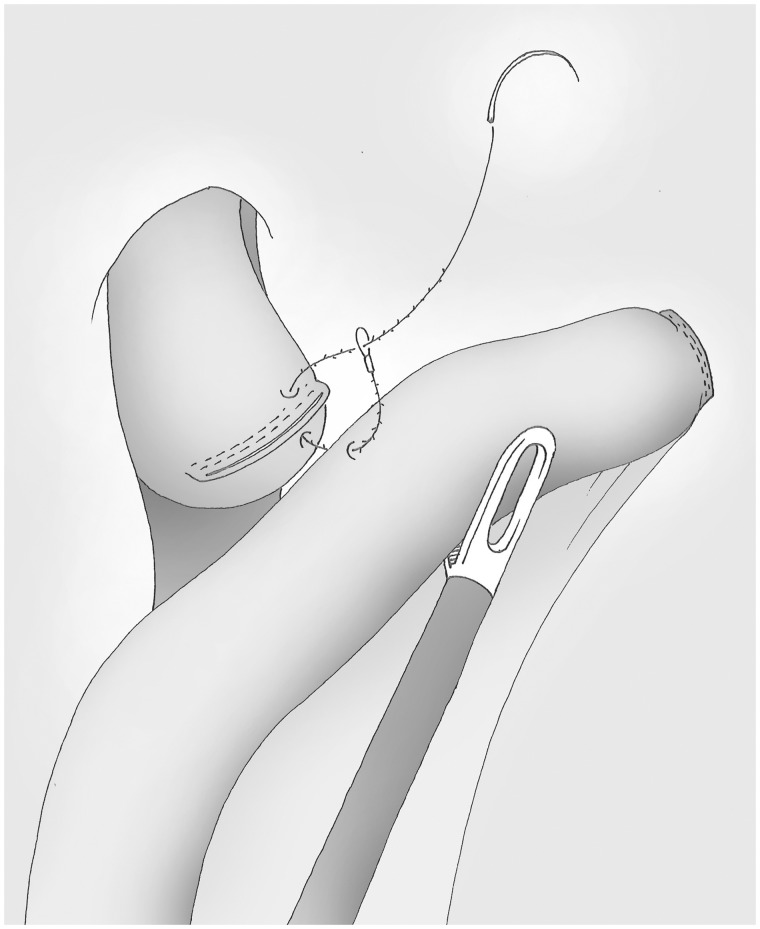
The oesophagus is maintained in the abdomen using a pressure on the Faucher tube and the jejunum is tracked to the left hypochondrium. The suture using a 15 cm V-loc® starts on the left and includes the oesophageal staple line.

### Oesophago-jejunal anastomosis (posterior layer) (Fig. 12)

The total plan is both jejunum and the oesophagus and taking the oesophageal staple line early in the suture. While the suture is tensioned, the operator on the right side exposes the edges of the digestive tract. When the suture is completed, the wire is cut without a knot, with a tail of 1.5 cm.

**Fig. 12:**
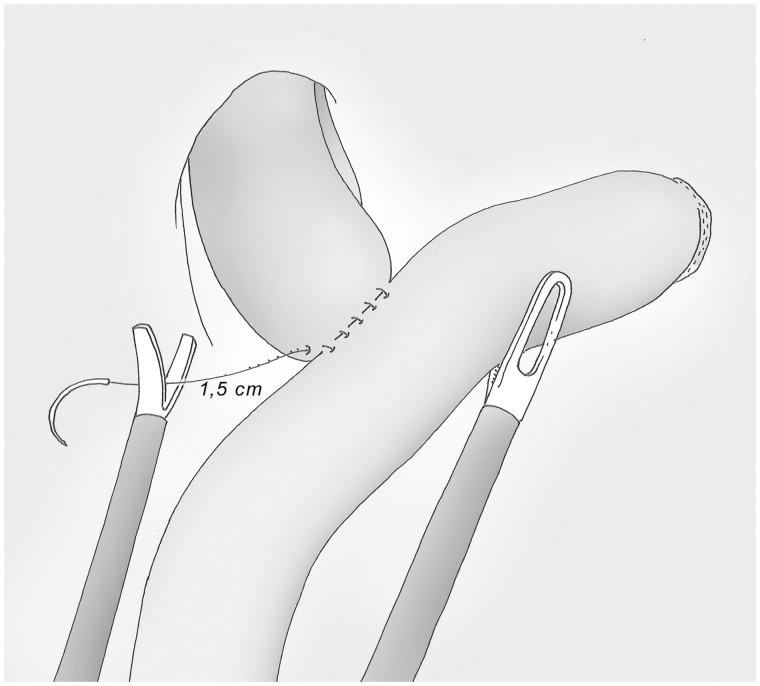
The posterior side of the anastomosis is made by a full layer, manual, running suture; the wire is cut without knot with a 1.5 cm tail.

### Opening the viscera (Fig. 13)

The small bowel is opened next to the posterior margin, leaving a few millimetres at both extremities, with a cautious haemostasis of the mucosae. The oesophagus is opened in the same way again with the help of the pressure of the Faucher tube, which can then be removed.

**Fig. 13:**
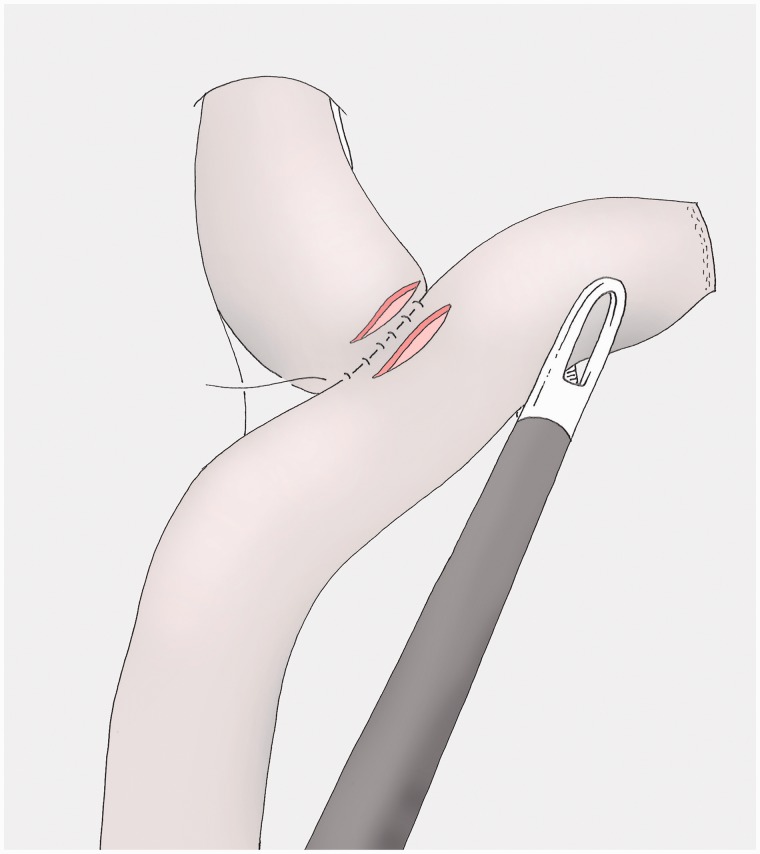
The viscera are opened, leaving a few millimetres at both extremities, with the help of a pressure on the Faucher tube for the oesophageal side.

### Reinforcement of the posterior layer (optional) (Fig. 14)

Simple resorbable stitches reinforce the posterior layer of the anastomosis, optimize the haemostasis and bind the protuberant intestinal mucosa.

**Fig. 14:**
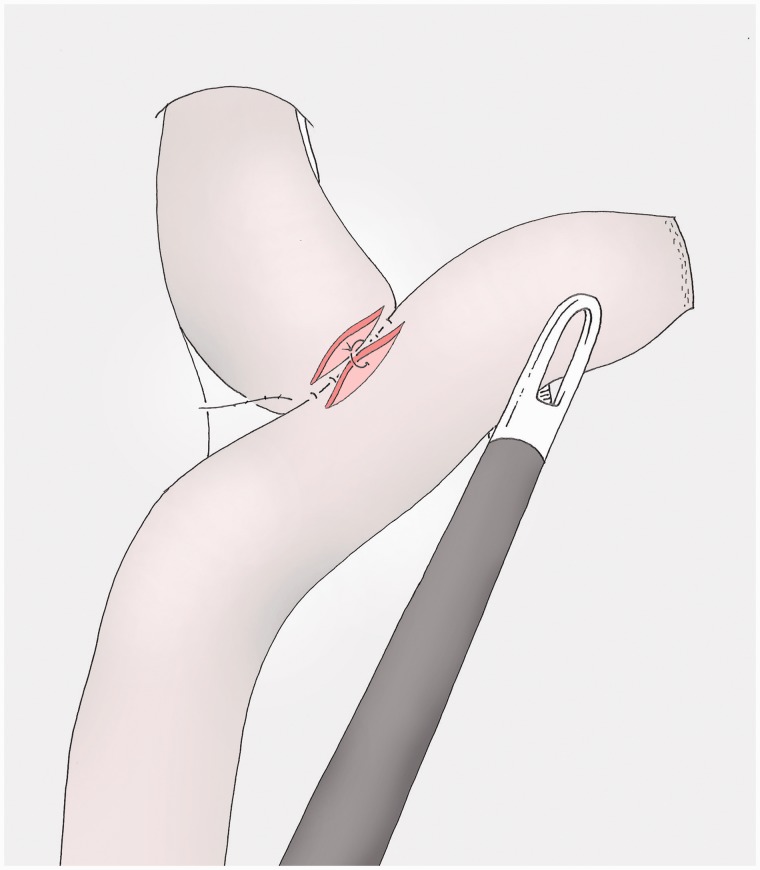
Optional stitches of the posterior side of the anastomosis, using absorbable material in full layer fashion.

### Oesophago-jejunal anastomosis (anterior layer) (Fig. 15)

The single layer suture using the 15 cm V-loc® starts from the left at the first stitch of the posterior suture and fixes the jejunum to the oesophagus with large extramucosal stitches in the jejunum and full-thickness stitches in the oesophageal wall. The oesophageal mucosa, which is the really solid layer of the oesophagus, does not retract due to the fact that the oesophagus is open after the completion of the posterior layer. The operator places an atraumatic clamp in the oesophagus to keep it open when it is necessary to pass through the oesophagus (usually done in reverse). In clinical practice, there is no need to insert a nasogastric tube but one can be inserted after the beginning of the suture [10]. The suture ends beyond the end of the posterior suture and the wire is then cut without a knot with a tail of 1.5 cm.

**Fig. 15:**
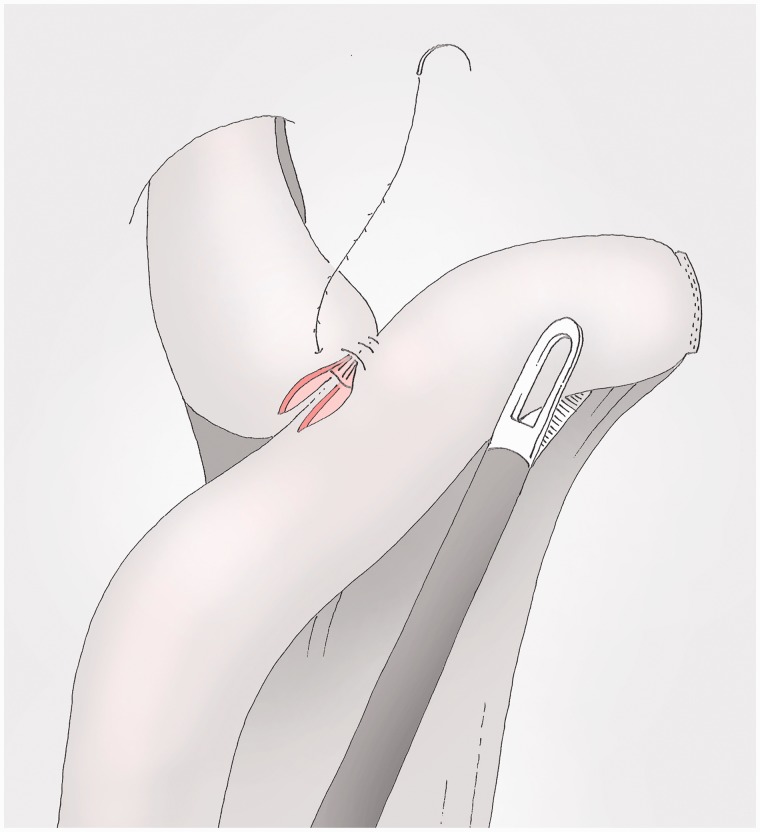
The anterior layer starts from the beginning of the posterior one and ends beyond the end of it. The wire is cut without knot with a 1.5 cm tail.

### Fixation of the alimentary loop and drainage (Fig. 16)

The top of the alimentary loop is attached to the pillar of the diaphragm. We do not systematically perform a blue- or air test; they are only done if the oesophageal mucosa is torn or if there is a need for addition anastomotic sutures. A retro-anastomotic Penrose drain covers the anastomosis and eventually a second one can be used for the duodenal stump. The operation ends with the removal of the specimen by a suprapubic incision.

**Fig. 16:**
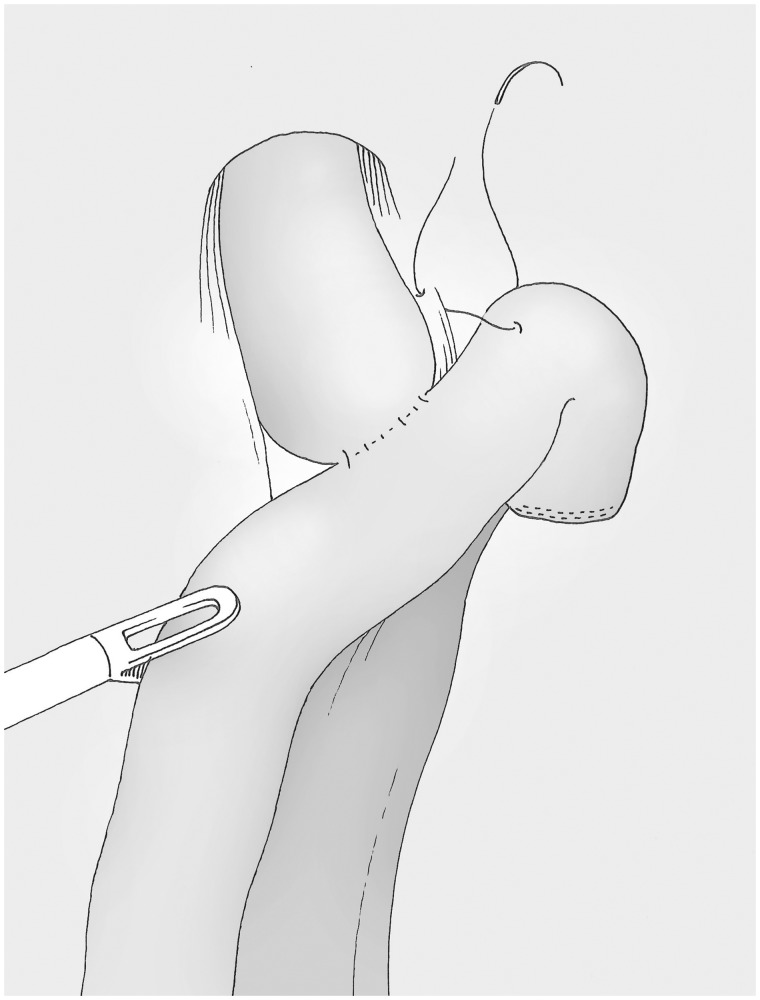
The alimentary loop is fixed to the pillar of the diaphragm.

## CONCLUSION

Totally laparoscopic total gastrectomy with D2 lymphadenectomy is a feasible and reproducible technique for locally advanced gastric cancer. Its advantages are the usual ones of the laparoscopic approach (significant reduction of intraoperative blood loss, improved postoperative recovery, avoiding unnecessary laparotomy, fewer wall complications and shorter hospital stay) [11]. The quality of the surgical resection seems to be equivalent to the standards achieved with laparotomy [2, 4, 12]. The major challenge is the oesophago-jejunal anastomosis which is performed in our department using two single layer V-loc® sutures since June 2011 without postoperative mortality and with a rate of anastomotic fistula less than 5%, always treated conservatively.

**Conflict of interest:** none declared.
